# Human Hepatocyte-Derived Induced Pluripotent Stem Cells: MYC Expression, Similarities to Human Germ Cell Tumors, and Safety Issues

**DOI:** 10.1155/2016/4370142

**Published:** 2016-01-06

**Authors:** Carmen Unzu, Marc Friedli, Alexis Bosman, Marisa E. Jaconi, Barbara E. Wildhaber, Anne-Laure Rougemont

**Affiliations:** ^1^Pediatric Surgery Laboratory, Department of Pathology and Immunology, Faculty of Medicine, University of Geneva, 1211 Geneva, Switzerland; ^2^School of Life Sciences and “Frontiers in Genetics” National Program, École Polytechnique Fédérale de Lausanne (EPFL), 1015 Lausanne, Switzerland; ^3^Department of Pathology and Immunology, Faculty of Medicine, University of Geneva, 1211 Geneva, Switzerland; ^4^Division of Clinical Pathology, Geneva University Hospitals, 1211 Geneva, Switzerland

## Abstract

Induced pluripotent stem cells (iPSC) are a most promising approach to the development of a hepatocyte transplantable mass sufficient to induce long-term correction of inherited liver metabolic diseases, thus avoiding liver transplantation. Their intrinsic self-renewal ability and potential to differentiate into any of the three germ layers identify iPSC as the most promising cell-based therapeutics, but also as drivers of tumor development. Teratoma development currently represents the gold standard to assess iPSC pluripotency. We analyzed the tumorigenic potential of iPSC generated from human hepatocytes (HEP-iPSC) and compared their immunohistochemical profiles to that of tumors developed from fibroblast and hematopoietic stem cell-derived iPSC. HEP-iPSC generated tumors significantly presented more malignant morphological features than reprogrammed fibroblasts or CD34+ iPSC. Moreover, the protooncogene* myc* showed the strongest expression in HEP-iPSC, compared to only faint expression in the other cell subsets. Random integration of transgenes and the use of potent protooncogenes such as* myc* might be a risk factor for malignant tumor development if hepatocytes are used for reprogramming. Nonviral vector delivery systems or reprogramming of cells obtained from less invasive harvesting methods would represent interesting options for future developments in stem cell-based approaches for liver metabolic diseases.

## 1. Introduction

Currently, the only treatment for inherited metabolic liver diseases with severe extrahepatic manifestations consists of liver transplantation (LT). Gene therapy of diseased hepatocytes followed by their autotransplantation is an alternative approach to LT, as they allow correction of the metabolic defect while avoiding immunosuppression and responding to the shortage of donor livers. Yet the major obstacle for a sufficient, long-term correction of the disease by this method is the insufficient amount of autotransplantable hepatocytes.

One strategy for increasing the hepatocyte transplantable mass would be the use of stem cells. Induced pluripotent stem cells (iPSC) are endowed with intrinsic self-renewal ability and the potential to differentiate into any of the three germ layers and can thus be used to increase the hepatocyte transplantable mass. However, the same properties that make iPSC the most promising avenue for increasing HEP mass also carry a risk of tumorigenicity.

In stem cell research, the gold standard assay for assessing cell pluripotency is teratoma formation in immunosuppressed mice. Teratomas are benign tumors characterized by their rapid growth* in vivo* and a haphazard mixture of somatic tissues at varying degrees of differentiation [1]. In fact, this assay is used to check the ability of iPSC to form the three germ layers* in vivo*. The presence of immature elements, and mostly of primitive neuroectodermal rosettes, defines immature teratoma. These lesions are potentially malignant. Primitive germ cell tumors consist of tumors that contain malignant germ cell elements other than teratoma, namely, dysgerminoma/seminoma, yolk sac tumor, embryonal carcinoma, and nongestational choriocarcinoma [2]. Teratomas derived from engrafted iPSC may show primitive neuroectodermal rosettes that may be accompanied by yolk sac elements.

The use of oncogenes such as* myc* during the reprogramming of human somatic cells to iPSC is of particular interest since not only is MYC a notable example of proteins known to interfere with normal cell differentiation, but also overexpression of MYC targets is observed in poorly differentiated and aggressive human tumors [3]. Moreover, an influence of the tissue of origin in teratoma-forming propensity has been shown [4].

Nevertheless, few studies have examined teratoma formation for preclinical safety [5]. According to some authors, yolk sac elements may have been overlooked and underreported and malignant embryonal carcinoma cells have rarely been described in xenografts derived from human iPSC [5, 6]. On the other hand, little is known about the profile of expression of tumoral markers* in vivo*. Thus, a better understanding of the biology of iPSC and cellular reprogramming will highly contribute to the development of new strategies for safe treatments.

It has been described that iPSC keep the epigenetic memory of the cells of origin [7]. Focusing on the translational application of iPSC for liver diseases, for example, cell transplantation for the treatment of inherited metabolic liver disease, human hepatocytes would thus presumably be the best cell source for reprogramming. Therefore, we analyzed the tumorigenic potential of iPSC generated from human primary hepatocytes and compared histological findings and immunohistochemical profiles to those of tumors developed from foreskin fibroblast- and hematopoietic stem cell-derived iPSC as more accessible sources of cells.

## 2. Materials and Methods

### 2.1. Cells

Primary human hepatocytes were obtained from patients treated in the Swiss Center for Liver Diseases in Children at the Geneva University Hospitals after having obtained their parents' written consent. Cells were extracted from the child's native liver after they had undergone total hepatectomy as previously described [8]. Ethical approval for the cell isolation was obtained from the institutional ethics committee (approval number 08-028). Human foreskin fibroblasts were obtained from ATCC biological resources center (Manassas, USA). CD34+ cells from human cord blood were obtained and prepared as previously described [9].

### 2.2. Human iPSC Reprogramming

The polycistronic excisable reprogramming vector STEMCCA (hereafter called OKSM) was kindly provided by Professor Mostoslavsky (Boston University, MA, USA) [10]. Lentiviral particles encoding Oct-4, Klf4, Sox2, and Myc were prepared as previously described in [8]. The lentiviral vector expressing POU5F1 (OCT4), KLF4, and SOX2 as a single polycistronic transcript (hereafter called OKS) was kindly provided by Professor Naldini [11].

5 × 10^5^ human primary hepatocytes were plated on Matrigel or collagen before being transduced with OKS or OKSM using a MOI of 20. The same numbers of human foreskin fibroblasts were transduced with OKSM at MOI of 20. After 5 days, cells were switched to mTeSR1 medium (STEMCELL Technologies) and grown until reprogrammed colonies emerged (~20 days). 2.5 × 10^5^ CD34+ cells were transduced with OKS using 100 HC-TU per cell. After 5 days, cells were switched to mTeSR1 medium and grown on a mouse fibroblast feeder layer until reprogrammed colonies emerged (~21 d). Individual human iPSC clones were then picked and expanded on Matrigel-coated plates in mTeSR1 medium.

### 2.3. Mouse iPSC Reprogramming

Mouse hepatocytes and hematopoietic stem cells were obtained from transgenic mice in which the four reprogramming transcription factors were stably integrated as a single doxycycline-inducible polycistronic cassette within the ubiquitously expressed* Rosa26* locus (*ROSA*)*26Sor *[12]. Hepatocytes and hematopoietic cells were isolated as previously described in [8, 9]. iPSC generation was induced upon addition of doxycycline (2 *μ*g/mL) during 12 days. iPSC clones were then manually picked and expanded on gelatin-coated plates in Glasgow MEM (SIGMA) 15% FBS supplemented with 5% NEAA (Gibco), 100 *μ*M b-mercaptoethanol, 1 mM sodium pyruvate (Gibco), and 1000 U/mL of LIF (leukemia inhibitory factor (Chemicon)).

### 2.4. iPSC Characterization

Expression of pluripotency markers was addressed by immunofluorescence. iPSC were plated on Matrigel-coated glass coverslips. Before staining, coverslips were rinsed once with PBS and fixed for 15 minutes with 4% paraformaldehyde (PFA). Cells were rinsed 3 times with PBS for 5 minutes, blocked with 3% bovine serum albumin (BSA), and permeabilized with 0.3% Triton 9 X-100 in PBS for 1 h. Primary antibodies at appropriate dilutions were incubated ON at 37°C in PBS containing 0.1% Tween-20 and 1% BSA. Secondary antibodies at appropriate dilutions were incubated for 1 hour at 37°C together with DAPI 1 : 400. Finally, cells were washed and mounted onto glass slides with Mowiol and left overnight in the dark at room temperature (RT) before observation under the microscope. The kit of primary antibodies StemLight Pluripotency Kit (Cell Signaling Technology), which includes antibodies against Oct4, Sox2, Nanog, SSEA4, and Tra-1-60, was used. Secondary antibodies were donkey anti-mouse Alexa Fluor 488 and donkey anti-rabbit Alexa Fluor 536 (Life Technologies). Images were visualized with a Zeiss Axiophot microscope (Carl Zeiss) equipped with an Axiocam camera (Carl Zeiss); confocal images were obtained using the LSM 510 laser scanning confocal microscope (Carl Zeiss).

Embryoid bodies from the different clones were generated using the AggreWell plates and following manufacturers' instructions (STEMCELL Technologies). Karyotype analysis in all the iPSC clones following the G-banding method was performed by the Service of Cytogenetics of the University of Geneva.

1 × 10^6^ cells from the different iPSC clones at passage 3 in 100 *μ*L of Matrigel-PBS mix (1 : 1) were injected in a subcutaneous or intratesticular location in NOD/SCID mice to induce teratoma formation. Human embryonic stem (hES) cells were injected as a control. Three animals were injected per clone. Mice were sacrificed after 3 to 8 weeks, according to tumor size and development. Tumors were fixed in 10% buffered formalin for 48 h.

### 2.5. Histology, Immunohistochemistry, and* In Situ* Hybridization

Histological analysis was performed to assess teratoma-forming potential and tumor components. Specimens were entirely submitted for histological analysis, after gross processing and paraffin-embedding. Hematoxylin and Eosin (H&E) stains were performed on 3 *μ*m thick sections of the paraffin-embedded tissue.

Immunohistochemistry was conducted on selected tumors. High temperature antigen retrieval was applied. Reactivity to primary antibodies was identified using the Ultravision detection system and DAB as the chromogen substrate (Thermo Fisher Scientific) for the following antibodies: *β*-catenin (Novocastra mouse monoclonal, clone 17C2; dilution 1 : 20); CD30 (Dako mouse monoclonal, clone Ber-H2; prediluted); pooled cytokeratins (CK): AE1/AE3 + CK8/18 (Dako mouse monoclonal and rabbit monoclonal; AE1/AE3 dilution 1 : 100, CK8/18 1 : 25); Glypican 3, GPC3 (Cell Marque mouse monoclonal, clone 1G12; dilution 1 : 20); sal-like protein 4, SALL4 (BioCare Medical mouse monoclonal, clone 6E3; 1 : 100 dilution). The Envision visualization system (Dako) and DAB as the chromogen substrate were used for the remaining antibodies used: Alpha Fetoprotein, AFP (Novocastra, clone C3 mouse monoclonal; dilution 1 : 750); Chorionic Gonadotropin, *β*-subunit, *β*-HCG (Dako rabbit polyclonal; dilution 1 : 20); MYC (Abcam rabbit monoclonal, clone Y69; dilution 1 : 50); OCT4 (Abcam mouse monoclonal, clone SEMGC; dilution 1 : 2); and Placental Alkaline Phosphatase, PLAP (Dako mouse monoclonal, clone 8A9; dilution 1 : 10).

Albumin* in situ* hybridization was performed as previously described [13]. Briefly, a PCR template coding for the nearly full-length human albumin was generated from the MGC clone containing the complete cDNA for the human protein, purchased from Invitrogen (Invitrogen, Carlsbad, USA).

### 2.6. Immunohistochemistry Antibodies and* In Situ* Hybridization Probe

#### 2.6.1. SALL4

SALL4, a zinc-finger transcription factor, is a master regulator of embryonal pluripotency. Expressed in ES cells, SALL4 interacts with other pluripotency-related transcription factors such as OCT4 and NANOG. Whereas SALL4 is expressed in various fetal tissues, in adults only normal spermatogonia display reactivity. SALL4 has been identified as a useful immunohistochemical marker for germ cell tumors, being strongly expressed in seminoma, embryonal carcinoma, and yolk sac tumor. Focal reactivity is seen in choriocarcinoma, and limited expression is observed in differentiated teratoma components [14].

#### 2.6.2. OCT4

OCT4 (OCT3/4, the product of the* POU5F1* gene) is a transcription factor expressed in primordial germ cells and ES cells and is the most crucial POU domain transcription factor responsible for the maintenance of pluripotency and self-renewal. OCT4 nuclear expression is observed in ES cells and primordial germ cells during normal embryogenesis [15]. In germ cell tumors, OCT4 immunohistochemical expression is 100% sensitive for seminoma/dysgerminoma and embryonal carcinoma, whereas it is negative in yolk sac tumor and choriocarcinoma. Reactivity has also been shown in the immature neuroepithelium of high-grade immature teratoma [16].

#### 2.6.3. MYC (CMYC)

The MYC protein,* per se* a relatively weak transcriptional activator, influences numerous genes encoding proteins involved in cell cycle regulation, cell growth, metabolism, ribosome biogenesis, protein synthesis, and mitochondrial function. Mitogenic stimulation of quiescent cells induces rapid increases in MYC levels. In cancer, frequent* MYC* deregulation may occur by chromosomal translocation or gene amplification. Deregulation may also occur at the level of MYC mRNA and protein expression or stability (reviewed in [17]).

In different non-Hodgkin's lymphoma subtypes, MYC protein expression shows good correlation with the presence of* MYC* genetic translocations and has also major clinical implications [18].

#### 2.6.4. CD30 and PLAP

Among other immunohistochemical markers used for embryonal carcinoma, CD30, a member of the tumor necrosis factor receptor family, shows reactivity in 93% to 100% of embryonal carcinomas [19]. PLAP occurs focally as a membranous and/or cytoplasmic staining in most embryonal carcinomas; staining may also be seen in approximately half the yolk sac tumors and choriocarcinomas.

#### 2.6.5. GPC3

GPC3 is a membrane-anchored member of the glypican family of heparin sulfate that may play a role in promoting embryonic cell growth and differentiation. Among germ cell tumors, GPC3 has been shown to be expressed in all yolk sac tumors, usually displaying a moderate-to-strong and diffuse pattern of staining. Choriocarcinoma is also consistently positive, with strong staining in the malignant syncytiotrophoblasts and weaker staining in the cytotrophoblasts. In about one-third of the cases, immature teratoma may show variable reactivity in the primitive stroma, neuroepithelium, fetal-type glands, primitive tubules, and cartilage anlage. GPC3 is rarely expressed in embryonal carcinoma [20].

#### 2.6.6. AFP

AFP, a major plasma protein produced by the yolk sac and liver during fetal life, shows reactivity in about 85% of yolk sac tumors; AFP may also be positive in scattered cells in embryonal carcinoma. Contrary to the diffuse GPC3 pattern of staining in yolk sac tumors, AFP reactivity is often focal [19]. AFP is also expressed in the normal secondary human yolk sac that also shows reactivity to GPC3 and SALL4.

#### 2.6.7. *β*-Catenin

*β*-catenin is a multifunction protein that acts as a component of the cell-cell adhesion apparatus. In the nucleus *β*-catenin acts as a coactivator of the transcription of T-cell transcription factor/lymphoid enhancer binding factor (TCF/LEF) target genes, allowing for the recruitment of factors required for chromatin remodeling and transcriptional activation. Immunohistochemical *β*-catenin nuclear accumulation is observed in various tumor models and indicates deregulation of the Wnt pathway.

#### 2.6.8. Albumin

Albumin synthesis occurs in the liver, and human fetuses synthesize endogenous albumin from early pregnancy on [21]. Therefore, albumin detection represents a marker of hepatocyte differentiation. Of note, the hepatoid variant of yolk sac tumor may also express albumin [22], again in line with a marker of differentiation.

## 3. Results

### 3.1. General Pathology Findings

**Figure 1:**
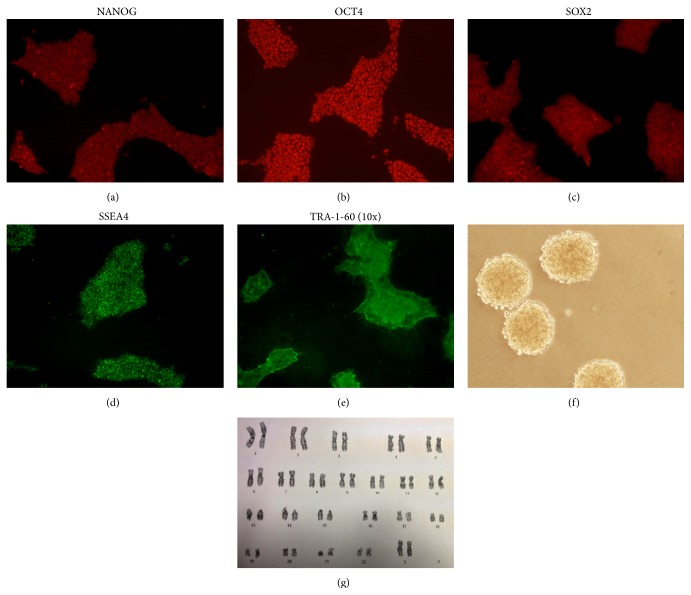
iPSC characterization. (a–e) Immunofluorescence shows the protein expression of the pluripotency markers NANOG, OCT4, SOX2, SSEA4, and TRA-1-60 (10x). (f) Embryoid body formation by the clones shows generation of the three germ layers* in vitro*. (g) Karyotyping showed no chromosomal aberration.

iPSC were generated from different human cell sources: hepatocytes, fibroblasts, and hematopoietic stem cells. Clones were characterized by immunofluorescence before injection, and expression of pluripotency markers was confirmed (Figures 1(a)–1(e)). The ability of the stable clones to generate the three germ layers* in vitro* was analyzed showing accurate embryoid body formation (Figure 1(f)). Besides, analysis of the karyotype of each clone was performed in order to discard any chromosomal aberration (Figure 1(g)). No differences among clones in the levels of pluripotent protein expression or embryoid body formation were observed.

The generated iPSC clones allowed for varying degrees of teratoma formation. The presence of immature, embryonal-type, or primitive tissue (generally neuroectodermal rosettes) identified immature teratoma. No dysplasia was seen in the teratoma epithelial components. Immunohistochemistry was performed to compare reactivity with known protein expression profiles in human germ cell tumors. Of note, no tumor displayed syncytiotrophoblastic cells, and *β*-HCG, a dimeric glycoprotein produced by placental trophoblastic cells and the main choriocarcinoma immunohistochemical marker, remained negative in all tumors assessed. Staining for *β*-catenin showed a membranous pattern of staining exclusively and no nuclear delocalization. Histological findings and main immunohistochemical results are summarized in Table 1.

**Figure 2:**
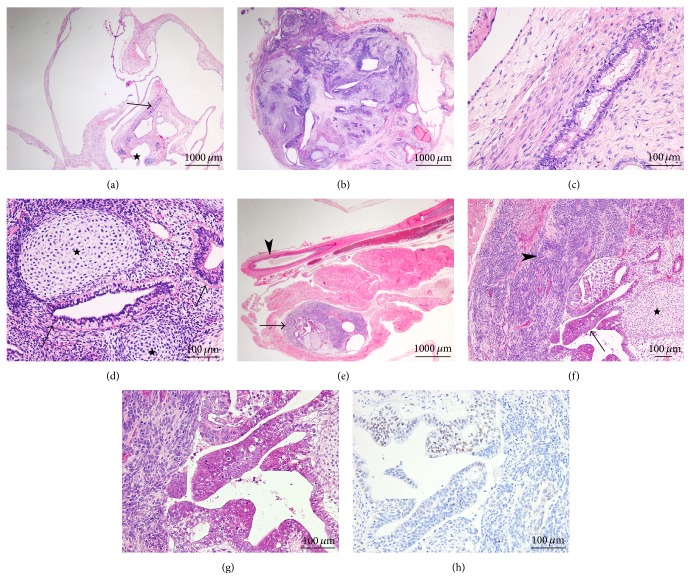
Human embryonic stem cells and teratoma formation. Teratomas generated were partly cystic (a) and partly solid (b) (Hematoxylin and Eosin (H&E), original magnification 20x). Tumors were composed of structures derived from all three germ layers; cystic glands were divided by paucicellular mesenchymal septa, in which a few slightly immature glandular structures were embedded (c, H&E 200x). Glands (*arrows*) and immature cartilage plates (*stars*) are shown as derivatives from the endodermal and mesodermal layers, respectively (d, H&E 200x). In one tumor, a small and solid 0.2 cm nodule (*arrow*) was located in an intramuscular position at a distance of 0.2 cm from the main cystic portion and close to the mouse vas deferens (*arrowhead*) (e, H&E 20x). The nodule was composed of immature neuroglial tissue (*arrowhead*), cartilage (*star*), and glands; a minority of the glandular structures were atypical (*arrow*) (f, H&E 100x). At higher magnification (g, H&E 200x), these glands were formed of cells showing nuclear atypia and increased apoptotic rate. Upon immunohistochemical evaluation, MYC reactivity was faint and focal, expressed in approximately 20% of the atypical cells (h, 200x).

hES cells injected as a control of teratoma formation in a subcutaneous and an intratesticular location generated as expected partly cystic and partly solid teratomas (Figures 2(a) and 2(b)), composed of structures derived from all three germ layers. Large cystic glands were separated by loose mesenchymal septa, in which a few slightly immature glandular structures were embedded (Figure 2(c)). Derivatives from the ectodermal layer (neuroectodermal rosettes and pigmented epithelium) and from the mesodermal layer (cartilage) were also seen (Figure 2(d)). In one tumor, a second portion consisted of a small and solid 0.2 cm nodule, located in an intramuscular position at a distance of 0.2 cm from the main cystic portion (Figure 2(e)). The nodule was composed of immature neuroglial tissue, cartilage, and glands (Figure 2(f)). A minority of the glandular structures showed nuclear atypia, increased apoptotic rate, but no mitoses (Figure 2(g)). MYC reactivity was however faint and focal, expressed in approximately 20% of the atypical cells (Figure 2(h)). Whereas SALL4 reactivity was strong, there was no reactivity to OCT4, CD30, or PLAP, thereby disproving embryonal carcinoma.

### 3.2. iPSC Generated from Human Hepatocytes

Seven clones were generated from human primary hepatocytes; five were subcutaneously injected and two in the mouse testis.

**Figure 3:**
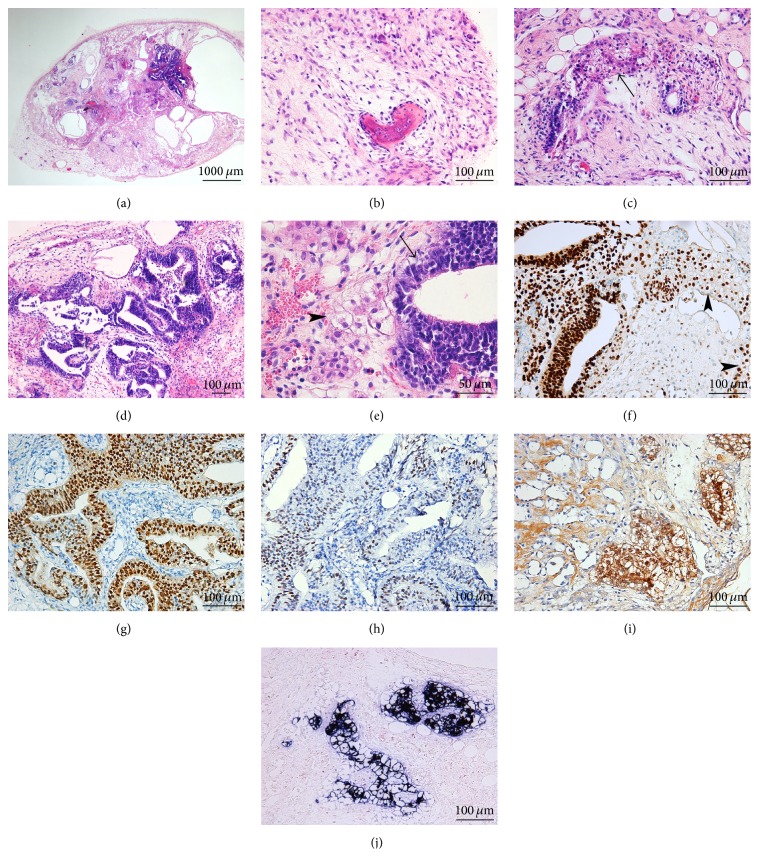
iPSC generated from human hepatocytes, using the OKS integrative vector. The tumors produced were in part cystic (a, H&E 20x) and showed features of immature teratoma. Fibrous bone was part of the mesodermal derivatives and was surrounded by loosely arranged mesenchymal cells (b, H&E 200x). Endoderm-derived structures consisted of variably immature glands focally in close relationship to fetal-like hepatocytes (*arrow*) (c, H&E 200x). Rarely, confluent atypical glandular structures showed stratification (d, H&E 100x); stratified glands (*arrow*) were also focally close to nests of fetal-like hepatocytes (*arrowhead*) (e, H&E 400x). Upon immunohistochemical evaluation, the atypical glands displayed strong expression of SALL4, a feature shared by some of the teratoma components of which the hepatocyte plates are (*arrowheads*) (f, 200x). Strong reactivity to OCT4 was also seen (g, 200x). MYC reactivity was only mild (h, 200x). The fetal-appearing hepatocytes expressed AFP (i, 200x) and albumin (j,* in situ* hybridization, 200x).

When hepatocytes were reprogrammed to iPSC using the OKS integrative vector, tumors were relatively small (up to 1 cm in greatest diameter) and either in part cystic (Figure 3(a)) or composed of loosely arranged tissues. No tumor development was observed after 9 weeks in the three mice injected with one of the OKS clones. Histologically, all three tumors from the second clone showed features of immature teratoma, with development of derivatives from all three primary germ layers. Ectodermal derivatives were mainly mature, with rare neuroectodermal rosettes in only one of the three tumors, representing less than 5% of the tumor volume. Mesodermal derivatives consisted of bone (Figure 3(b)), adipose tissue, and mesenchymal cells at varying degrees of maturation. Endoderm-derived structures consisted of variably immature glands and fetal-type hepatocytes (Figure 3(c)). In one tumor, confluent atypical glandular structures showed stratification. The cells displayed a high nucleocytoplasmic ratio, with large and hyperchromatic nuclei; apoptotic bodies were numerous, and minute foci of necrosis were seen (Figure 3(d)); stratified glands were also focally close to nests of fetal-like hepatocytes (Figure 3(e)). Upon immunohistochemical evaluation, pooled cytokeratins highlighted the epithelial structures and showed more moderate reactivity in the atypical glands. The latter also strongly expressed SALL4 and OCT4 (Figures 3(f) and 3(g)). Mild reactivity was seen for MYC (Figure 3(h)) and CD30, whereas PLAP remained negative. Epithelial structures and a minority of the atypical glands (20%) showed strong GPC3 reactivity. The fetal-appearing hepatocytes expressed AFP (Figure 3(i)) and GPC3 and were also decorated by albumin (Figure 3(j)).

**Figure 4:**
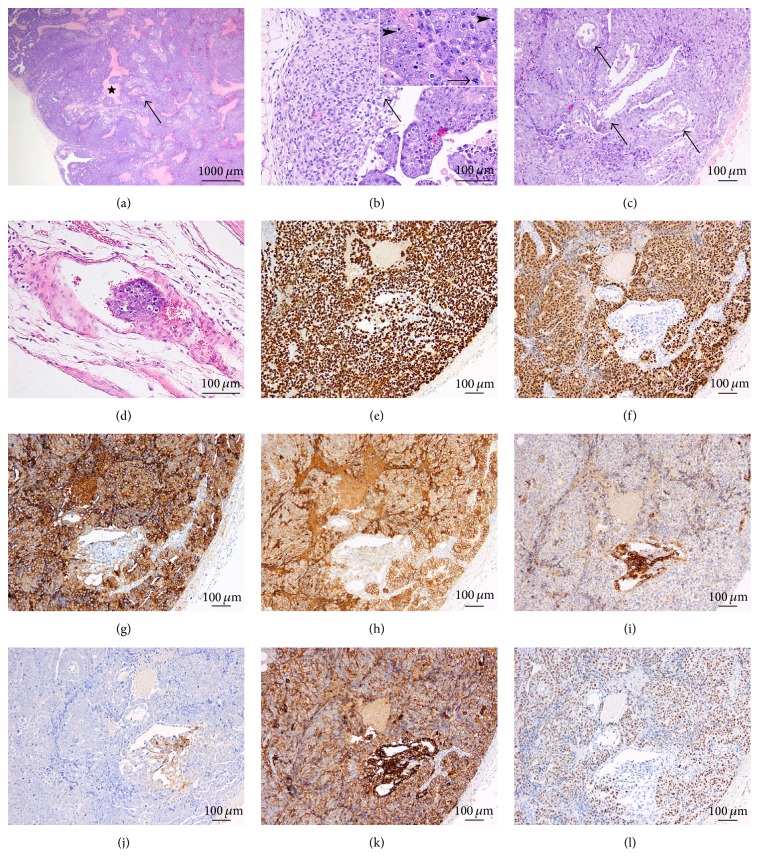
iPSC generated from human hepatocytes, using the OKSM integrative vector. The generated tumors were mostly solid, with an intimately admixed minor component of irregular gland-like spaces (*arrow*); tumor cells formed serpiginous ribbons around central foci of tumor necrosis (*star*) (a, H&E 20x). Rarely, minute foci of immature mesenchymal cells were seen (*arrow*) (b). The major component was composed of pleomorphic malignant-appearing embryonal carcinoma cells that were large and primitive-appearing, with vesicular nuclei and prominent nucleoli; mitoses (*arrow*) and apoptotic bodies (*arrowheads*) were numerous (b, inset, H&E 400x). Gland-like spaces lined by immature cells were focally seen and corresponded to a minor yolk sac tumor component (*arrows*) (c, H&E 100x). Intravascular tumor embolism from the embryonal carcinoma component, a further malignancy criterion, was seen (d, H&E 200x). In the embryonal carcinoma component, strong and diffuse nuclear expression of SALL4 (e, 200x) and OCT4 (f, 200x) was seen, as well as moderate-to-strong membranous and cytoplasmic PLAP (g, 200x), and CD30 (h, 200x) reactivity. Contrariwise, the yolk sac component showed strong GPC3 (i, 200x) and AFP (j, 200x) reactivity. Pooled cytokeratins were expressed in both components but strongly in the yolk sac glandular structures compared to in the embryonal carcinoma component (k, 200x). MYC nuclear reactivity was seen exclusively in the embryonal carcinoma cells (l, 200x).

Most of the clones reprogrammed with the OKSM lentiviral vector produced tumors composed of a major embryonal carcinoma-like component (5/6). Three of four clones injected subcutaneously induced solid tumors larger than 1 cm in less than 4  weeks. All displayed malignant histological features, being composed nearly exclusively (95% or more) of an embryonal carcinoma-like component. The pattern of growth was mostly solid, with an intimately admixed minor component of irregular gland-like spaces (Figure 4(a)). Tumor cells formed serpiginous ribbons around central foci of tumor necrosis and were focally lined by fibrovascular septa or by minute foci of immature mesenchymal cells (Figure 4(b)). Tumor cells were large and primitive-appearing, with vesicular nuclei and prominent nucleoli (Figure 4(b), inset). Mitoses, some of which are atypical, and apoptotic bodies were numerous. Intravascular tumor embolism was seen (Figure 4(c)). Gland-like spaces formed a minor tumor component (Figure 4(d)). Nuclear expression of SALL4 (Figure 4(e)) and of the pluripotency marker OCT4 (Figure 4(f)) was strong and diffuse, whereas membranous and cytoplasmic PLAP reactivity (Figure 4(g)) was seen in 70% or more of the cells, with varying intensity. Membranous staining for CD30 was moderate to strong (Figure 4(h)). However, nests of negative cells were highlighted by this staining, appearing even more primitive and rounded. Contrary to the embryonal carcinoma cells, these cells forming gland-like spaces expressed GPC3 (Figure 4(i)) and AFP (Figure 4(j)) and showed stronger reactivity for pooled cytokeratins than the embryonal carcinoma component (Figure 4(k)). The morphological features and the immunohistochemical pattern of expression were consistent with a minor yolk sac tumor component. Moderate-to-strong MYC nuclear reactivity (Figure 4(l)) was seen only in the embryonal carcinoma cells.

The two hepatocyte-derived clones injected in the testis also produced large tumors. One of the tumors was composed nearly exclusively of embryonal carcinoma, with only minute foci of immature mesenchyme. The second clone produced mixed germ cell tumors, made of teratoma and embryonal carcinoma, the latter representing 40 to 60% of the tumor. Immunohistochemical profile in the embryonal component was similar to the clones injected subcutaneously, except for mild GPC3 staining and absence of AFP reactivity. Immature teratoma was also seen in the tumors generated by this second clone, with derivatives from the three germ layers; however, no liver differentiation was seen.

The only clone differing from the above-mentioned clones was injected subcutaneously and produced partly cystic and partly solid tumors, measuring up to 1 cm in greatest diameter after 6 weeks. The tumors corresponded to mixed germ cell tumors with immature teratoma, embryonal carcinoma, and small foci of yolk sac tumor. The immature teratoma component contained derivatives from all three germ layers, of which neuroectodermal rosettes represented around 10%. Another 10% of the tumors were composed of embryonal carcinoma, strongly reactive to SALL4, OCT4, and PLAP, and showing moderate expression of CD30. GPC3 reactivity was mild and focal. MYC staining was strong.

### 3.3. iPSC Generated from Human Fibroblasts

**Figure 5:**
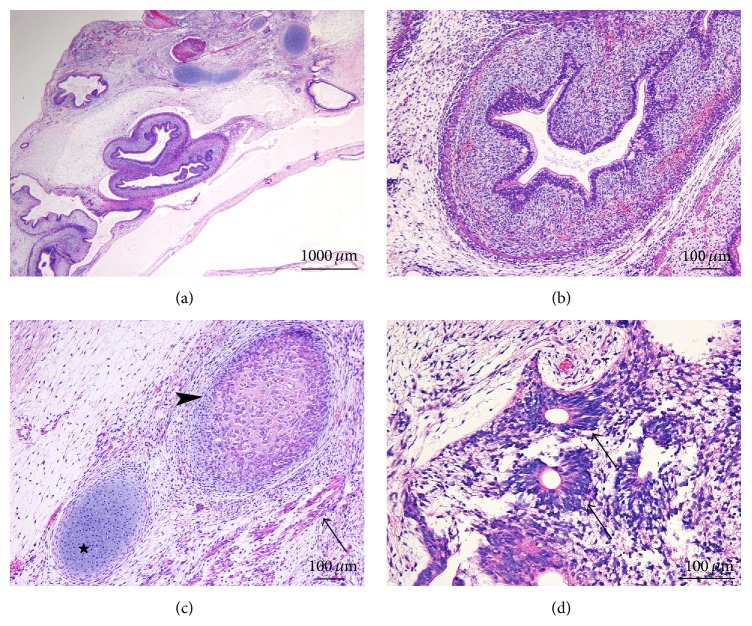
iPSC generated from human fibroblasts. The tumors generated were mainly cystic (a, H&E 20x), and derivatives from all three germ cell layers were seen. Intestinal type structures were lined by a mucin producing epithelium, overlying a well-differentiated wall with a smooth muscle layer (b, H&E 100x). Mesodermal derivatives also consisted of immature bone (osteoid) (*arrowhead*) and cartilage (*star*), as well as striated muscle (*arrow*) (c, H&E 100x). Ectodermal derivatives corresponded mainly to immature neuroglial tissue and neuroectodermal rosettes (*arrows*) (d, H&E 200x).

All the tumors from fibroblast-derived clones were mainly cystic (Figure 5(a)). Derivatives from all three germ cell layers were seen (Figures 5(b) and 5(c)), the ectodermal derivatives corresponding mainly to immature neuroglial tissue and neuroectodermal rosettes (Figure 5(d)), identified as a minor component (with a maximum of 10%). No embryonal carcinoma-like foci were seen. No differences were observed between the tumors injected subcutaneously and in the testis.

### 3.4. iPSC Generated from CD34+ Hematopoietic Stem Cells

**Figure 6:**
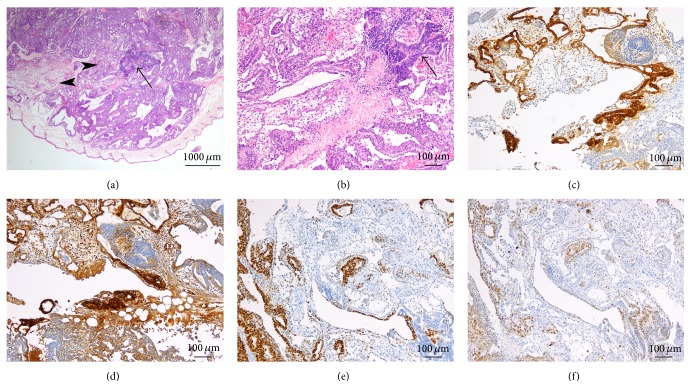
iPSC generated from CD34+ hematopoietic stem cells. One of the three clones produced a partly solid tumor mainly composed (95%) of an embryonal carcinoma component (a, H&E 20x), with only rare neuroectodermal rosettes (*arrow*) and a minor yolk sac component (*arrowheads*). The yolk sac component consisted in a loose meshwork of primitive-appearing cells lining cystic spaces (*arrow*: neuroectodermal rosettes) (b, H&E 100x). Whereas strong reactivity for GPC3 (c, 200x) and AFP (d, 200x) was seen in the yolk sac tumor component, reactivity for OCT4 (e, 200x) highlighted the embryonal carcinoma component only. MYC staining was moderate in the embryonal carcinoma cells, whereas no reactivity was seen in the yolk sac component (f, 200x).

All the clones derived from CD34+ hematopoietic stem cells were generated with the OKS lentiviral vector and subcutaneously injected. Tumors were immature teratoma, with derivatives from the 3 germ layers in 2/3 of the cases. A third clone produced a more solid tumor (Figure 6(a)), with only rare neuroectodermal rosettes, and minute foci of immature mesenchymal cells; primitive structures were reminiscent of endodermal sinus. This yolk sac component was composed of a loose meshwork of primitive-appearing cells lining cystic spaces (Figure 6(b)). More than 90% of the tumor was composed of pluristratified glandular and vesicular structures, showing nuclear atypia, and high apoptotic and mitotic rates; small foci of necrosis were seen. SALL4 showed strong and diffuse staining; the small focus with neuroectodermal rosettes was however lost on the slide used for this marker. Strong albeit focal reactivity for GPC3 (Figure 6(c)) and AFP (Figure 6(d)) was seen in the yolk sac tumor component. Pooled cytokeratins were negative in the neuroectodermal rosettes and expressed in the epithelial structures with varying intensities, as well as in some spindle cells in the mesenchymal septa. Reactivity for OCT4 (Figure 6(e)) highlighted the embryonal carcinoma foci but was negative in both the endodermal sinus derivatives (yolk sac tumor foci) and the neuroectodermal rosettes. A minority of the embryonal carcinoma cells expressed CD30 and PLAP, whereas MYC staining was moderate in the embryonal carcinoma cells and mild and focal in the neuroectodermal rosettes; no reactivity was seen in the endodermal sinus structures (Figure 6(f)). The differential MYC activation and expression in this clone reprogrammed with the lentiviral vector encoding only Oct4, Sox2, and Klf4 could be due to insertional mutagenesis.

### 3.5. iPSC Generated from 4F2A Transgenic Mouse Cells

In order to evaluate the effect of insertional mutagenesis of the reprogramming transgene, additional clones were derived from mouse hepatocytes and hematopoietic stem cells from the transgenic 4F2A mice harboring the classic four transcription factors stably integrated as a single doxycycline-inducible polycistronic cassette within the ubiquitously expressed* Rosa26* locus.

**Figure 7:**
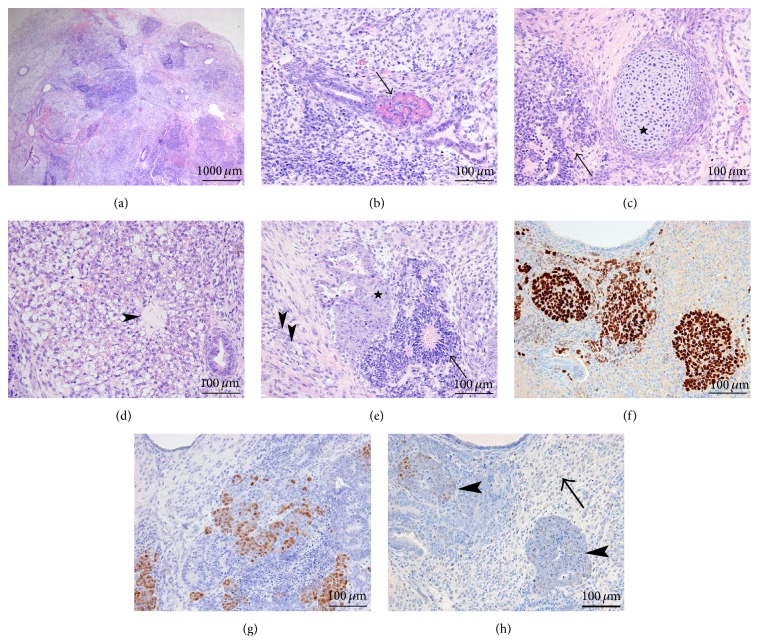
iPSC generated from mouse hepatocytes. All iPSC with mouse hepatic origin produced solid mixed germ cell tumors (a, H&E 20x). The teratoma components showed derivatives from all three germ layers, with development among other endodermal structures of ducts and pancreas (*arrow*) (b, H&E 200x), of cartilage as mesodermal derivative (*star*) (c, H&E 200x) and neuroglial tissue as ectodermal derivative (*arrow*) (c). Fetal-type hepatocyte plates were focally radially arranged around a centrilobular-like vein (*arrowhead*) (d, H&E 200x). A minor embryonal carcinoma component was composed of tumor cells arranged in cords and gland-like structures and forming small dispersed nests (*star*) within the neuroglial component (*arrow*); ganglion-like cells (*arrowheads*) are also seen (e, H&E 200x). Strong nuclear reactivity to SALL4 (f, 200x) and OCT4 (g, 200x) was seen in the embryonal carcinoma cells. MYC nuclear reactivity was faint and focal in the embryonal carcinoma component and moderate only in less than 1% of the cells (*arrowheads*); a minority of the neuroglial cells (*arrow*) also showed very faint nuclear staining (h, 200x).

All iPSC from hepatic origin produced mixed germ cell tumors with only minor embryonal carcinoma components (5% to 10%) together with teratoma, showing derivatives from all three germ layers (Figures 7(a)–7(c)). Small foci of fetal-type hepatocyte plates were radially arranged around a vein, reminiscent of pericentrilobular vein architecture (Figure 7(d)). The embryonal carcinoma cells were arranged in cords and gland-like structures, forming small dispersed nests within the teratoma component (Figure 7(e)). Strong nuclear reactivity was seen for SALL4 (Figure 7(f)), whereas OCT4 reactivity was moderate and focal (Figure 7(g)). A striking difference with the human-derived cell lines was that MYC nuclear reactivity was faint in approximately 30% of the cells and moderate in rare nuclei (less than 1%); very faint nuclear staining was also observed in a minority of the neuroglial cells, in the abundant glial component (Figure 7(h)). AE1/AE3 keratin reactivity was confined to the more differentiated epithelial structures.

**Figure 8:**
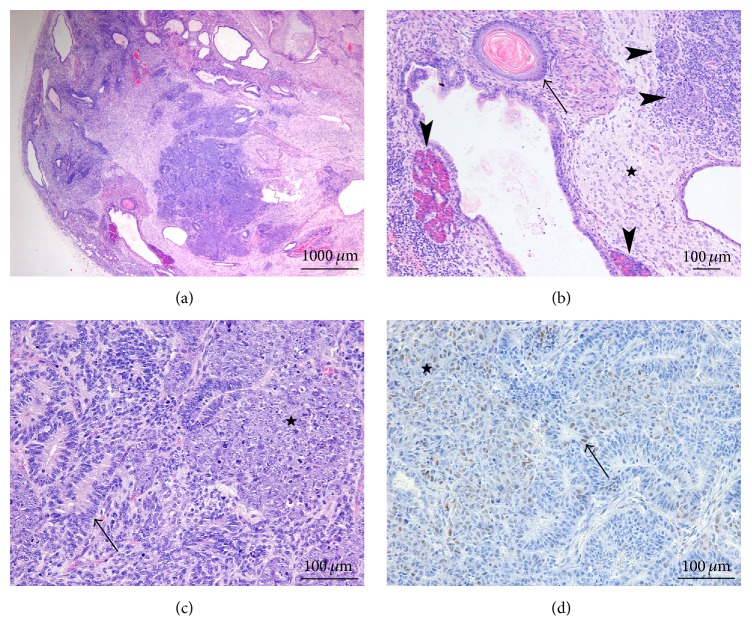
iPSC generated from mouse hematopoietic stem cells. Tumors produced were mainly solid mixed germ cell tumors (a, H&E 20x). The teratoma component showed derivatives from all three germ layers (b, H&E 100x), with the ectodermal (neuroglial,* star*) and endodermal (squamous epithelium,* arrow*, and exocrine pancreas,* vertical arrowheads*) derivatives being shown; small foci of embryonal carcinoma (*horizontal arrowheads*) were intermingled. At higher magnification (c, H&E 200x), the embryonal carcinoma cells formed small dispersed nests (*star*) within the teratoma component (immature neuroglial tissue,* arrow*). MYC immunohistochemical reactivity was very faint and focal in the embryonal carcinoma foci (d, 200x).

Mouse hematopoietic stem cell-derived clones produced large solid tumors, consisting in immature teratomas, with structures derived from all three germ layers (Figures 8(a) and 8(b)). Neuroglia was usually abundant and more mature than in the other tumor models. Endodermal derived structures were also more differentiated. One clone also produced minute foci of yolk sac tumor, and all three clones showed a minor embryonal carcinoma component (representing 5% to 10% of the tumor volume), often closely related to immature neuroglial tissue (Figure 8(c)). However, MYC immunohistochemical reactivity was also faint and focal in these embryonal carcinoma foci (Figure 8(d)).

## 4. Discussion

iPSC not only offer a promising source of pluripotent cells for regenerative medicine purposes, but also provide insights in the tumorigenic mechanisms of germ cell tumors, a tumor model associated with particular developmental-like differentiation processes.

In the 2014 WHO Classification of Tumours of Female Reproductive Organs [2], germ cell tumors comprise dysgerminoma, yolk sac tumor, embryonal carcinoma, nongestational choriocarcinoma, mature teratoma, immature teratoma, and mixed germ cell tumors. In the testis, the teratoma category also comprises dermoid cyst, monodermal teratoma, and teratoma with somatic type malignancies; immature teratoma is not a recognized category. Tumors showing mixed features are considered as tumors of more than one histological type or mixed forms [23]. Yolk sac tumor and choriocarcinoma resemble extraembryonic tissues [24].

Differences in nomenclature between the clinical and fundamental research settings may be confusing. A human testicular or ovarian tumor associating mixed embryonal carcinoma and teratoma would be considered a mixed germ cell tumor, whereas a similar tumor would be called teratocarcinoma in a murine setting [25]. In an attempt to clarify the confusion in terminology, the position by* Nature Biotechnology* was to consider “teratocarcinoma” as a malignant tumor composed of both somatic tissues and undifferentiated embryonal carcinoma cells [26].

### 4.1. General Features of iPSC Tumors

Teratoma formation after iPSC transplantation is thought to result from residual undifferentiated cells contained within the transplanted cells [27]. On the other hand, embryonal carcinoma cells represent the multipotential and undifferentiated malignant stem cells of “teratocarcinoma” [28]. Under nonneoplastic conditions, they can undergo self-renewal or differentiate into multiple mature cells types, a feature shared by ES cells. Interestingly, most of the reprogrammed iPSC clones in our study showed at least 5% of residual undifferentiated cells, which means that they are mixed germ cell tumors. Unfortunately, this pathological description of the iPSC is often underreported due to the fact that researchers normally look for histological structures of differentiated cells when characterizing the clones. For their use as an alternative to liver transplantation in inborn liver metabolic diseases, iPSC will be differentiated to hepatocytes before transplantation. However, differentiation protocols are not 100% efficient and even though it has been described that embryonal carcinoma cell lines have limited growth, we should keep in mind this percentage of malignant potential. Furthermore, similar to a majority of human embryonal carcinomas, the stem cells of human embryonal carcinoma cells express CD30, a feature that has been clearly associated with decreased levels of apoptosis [29]. Induction of karyotype abnormalities in hES cells, with gain of an extra copy of chromosome 12, is paralleled with the acquisition of CD30 expression and the generation of teratomas exhibiting a more primitive, undifferentiated phenotype [29].

### 4.2. OKSM-Derived iPSC Tumors

In those iPSC clones generated from hepatocytes with the OKSM lentiviral vector, the embryonal carcinomas were completely solid and undifferentiated and tumors did not show other differentiated components. Only one clone of that subset generated a teratoma with 5–10% of expected residual undifferentiated cells. This striking result suggests that hepatocytes in particular are more sensitive to the induction reprogramming than other cells like fibroblasts or hematopoietic cells, probably due to the fact that under physiological conditions they are quiescent cells that rarely divide. The adult human liver however maintains a certain amount of plasticity, the hepatocytes located in zones 1 and 2 of the liver lobule being, for instance, capable of undergoing biliary reprogramming after injury [30]. Moreover, we have shown here that malignant features were more closely related to the influence of one of the transgenes, the* myc* protooncogene.

### 4.3. The Influence of* myc*

MYC is at the crossroads of many important biological pathways and processes involved in neoplastic cell growth and proliferation, promoting both iPSC generation and carcinogenesis. In the developing human gonad, germ cell differentiation, proliferation, and apoptosis must remain tightly controlled, in order to avoid the malignant proliferation of stem cells. Oncogene and tumor suppressor gene interplay is crucial. ES cells, carcinoma* in situ* [31], and embryonal carcinoma all display similarities in gene expression profiles [32], consistent with a limited window of transformation from primitive germ cells/gonocytes to dormant precarcinoma* in situ* cells [33].

Previously, malignancy emerging from iPSC (ganglioneuroblastoma and follicular carcinoma of the thyroid) has been attributed to* myc* retrovirus reactivation, leading the authors to suggest replacement of the retrovirus-mediated system by an adenovirus-mediated system, allowing transient expression [34]. Here we further confirm that the generation of pure embryonal carcinoma is related to the use of* myc* during reprogramming. This link is stronger in hepatocyte-derived iPSC and correlates with MYC expression in terms of both staining intensity and percentage of positive cells, as assessed by immunohistochemistry.

The tumors produced from hepatocyte-derived iPSC reprogrammed without* myc* showed a minor embryonal carcinoma component (~10%), with only mild MYC immunohistochemical expression. The latter finding might be indicative of a more advanced stage of differentiation, since terminal differentiation results in decreased MYC expression [25]. Of note, small foci of fetal hepatocytes were part of the teratoma elements. This was also the case when* myc* was stably integrated in the transgenic 4F2A mice, confirming that insertional mutagenesis is also a triggering factor.

For instance, one of the three CD34+ hematopoietic stem cell-derived clones reprogrammed with the OKS lentiviral vector produced tumors with high malignant content, consisting of embryonal carcinoma and yolk sac tumor. In this case MYC reactivity was moderate in the embryonal carcinoma component; since* myc* was not included in the reprogramming vector, we believed these findings to be related to insertional mutagenesis.

iPSC derived from 4F2A mouse hepatocytes and hematopoietic cells that had stably integrated the OKSM transgene at the* Rosa26* locus allowed teratoma formation with only low levels of malignant structures, embryonal carcinoma foci representing 5% to 10% of the tumor volume. MYC immunohistochemical positivity was only focal, mostly faint, and only exceptionally moderate. No differences in tumor formation were seen between iPSC derived from mouse hepatocytes and hematopoietic cells. Other members of the* Myc* family of genes, and more specifically* l-myc*, promote human iPSC generation more effectively than* c-myc* and with weaker malignant transformation activity [35]. Use of alternative nonintegrative or integration-directed transgenes may therefore be beneficial for future clinical applications of iPSC technologies.

Of note, incompletely reprogrammed, early-passage iPSC that have not completely downregulated the expression of the lentiviral vector encoded transcription factors used during reprogramming (Nanog, Lin28, Sox2, and Oct4) have been shown to generate malignant germ cell-like tumors [6]. The presence of yolk sac tumor or embryonal carcinoma was attributed to the reactivation of one or more transgenes used during reprogramming [6]. We observed similar findings in three clones generated from human fibroblasts injected after the first passage (*data not shown*) when the OKSM lentiviral vector had been used for reprogramming. The tumors generated were exclusively composed of embryonal carcinoma-like foci. Immunohistochemical reactivity was similar to that described for the clones generated from the reprogramming of human hepatocytes, except for one tumor, which further showed a partly nodular pattern of growth. In the nodules only, despite strong SALL4, OCT4, and MYC reactivity, pooled cytokeratins, CD30, and PLAP remained entirely negative. These findings were suggestive of an even more primordial and undifferentiated cell state within the nodules.

## 5. Conclusion

Since iPSC keep the epigenetic memory of the cells of origin, human hepatocytes would presumably be the best cell source for potential therapeutic application in human liver diseases. Yet we showed that clones derived from human hepatocytes tend to produce tumors with malignant morphological features, including the propensity to develop metastasis as exemplified by an intravascular tumor embolism. This was not the case when fibroblasts or hematopoietic cells were reprogrammed or when* myc* transgene was stably integrated in the 4F2A mice cells. These findings suggest that random integration of transgenes and the use of potent protooncogenes such as* myc* would represent a greater risk of malignant tumor development if hepatocytes are used for reprogramming. Therefore nonviral vector delivery systems or reprogramming of cells obtained from less invasive harvesting methods would represent interesting options for future developments in stem cell-based approaches for liver metabolic diseases.

## Figures and Tables

**Table 1:** Main histological findings and immunohistochemistry profile in the tumors generated.

Cell origin	Clone	*myc* during reprogramming	Teratoma			Malignant GCT		Immunohistochemistry						
			Ectoderm	Mesoderm	Endoderm	EC	YST	MYC	SALL4	OCT4	CD30	PLAP	GPC3	AFP
Human ESC	ES 5	No	NE rosettes, pigmented epithelium	Cartilage	Glands	—	—	ND	ND	ND	ND	ND	ND	ND
Human ESC	S053	No	NE rosettes, pigmented epithelium	Cartilage	Glands	<5% EC-like	—	30% EC-like 1+	EC-like 3+ 10% TE 1+	Neg.	Neg.	Neg.	ND	ND
Human hepatocytes	OKS1	No	Nerves, rare NE rosettes	Bone, adipose tissue	Glands, 10% liver	10%	—	60% EC 1+	EC 3+ 10% TE 3+	EC 3+	EC 1+	Neg.	20% EC 1+ 30% TE 3+	Fetal hepatocytes 2+
Human hepatocytes	OKSM11	Yes	—	—	—	100%	—	Diffuse 2+	Diffuse 3+	90% 3+	90% 3+	90% 2+/3+	5% 3+	Neg.
Human hepatocytes	OKSM12	Yes	—	Minute foci immature mesenchyme	—	95%	5%	Diffuse 1+/2+	Diffuse 3+	90% 3+	90% 2+/3+	70% 2+/3+	YST 3+	YST 3+
Human hepatocytes	OKSM2	Yes	—	—	—	100%	—	Diffuse 3+	Diffuse 3+	Diffuse 3+	30% 2+	60% 2+	2+ 10%	Neg.
Human hepatocytes	OKSM5	Yes	NE rosettes, pigmented epithelium	Bone, cartilage	Glands	10%	5%	EC 3+	EC & YST 3+ 10% TE 2+	EC 3+	EC 2+/3+	EC 3+	YST 3+	YST 3+
Human hepatocytes	IH1-1	Yes	—	Foci immature mesenchyme	—	~100%	—	ND	ND	ND	ND	ND	ND	ND
Human hepatocytes	IH5-1B	Yes	Rare NE rosettes, pigmented epithelium	Bone, cartilage	Glands	40%–60%	—	EC 2+/3+	EC 3+	EC 2+	70% EC 2+	90% EC 2+/3+	20% EC 1+ 5% TE 2+	Neg.
Human fibroblasts	iFS21	Yes	Very rare NE rosettes	Smooth muscle	Glands	—	—	ND	ND	ND	ND	ND	ND	ND
Human fibroblasts	iFS22	Yes	Very rare NE rosettes, immature neuroglia	Smooth muscle	Digestive tract segments	—	—	ND	ND	ND	ND	ND	ND	ND
Human fibroblasts	ICCL	Yes	NE rosettes, neuroglia	Smooth muscle, cartilage	Glands	—	—	ND	ND	ND	ND	ND	ND	ND
Human CD34+ cells	iPS 2	No	NE rosettes, neuroglia	Cartilage	Glands	—	—	ND	ND	ND	ND	ND	ND	ND
Human CD34+ cells	iPS 6	No	Rare NE rosettes	Minute foci immature mesenchyme	—	>90%	5%	EC 1+/2+	EC & YST 3+	EC 3+	40% EC 1+	20% EC 2+	YST 3+	YST 3+
Human CD34+ cells	iPS 43	No	NE rosettes	Mesenchymal cells	Glands	—	—	ND	ND	ND	ND	ND	ND	ND
Mouse hepatocytes	hep1 p10	Stably integrated	Neuroglia	Smooth muscle	Pancreas, glands, squamous epithelium	5%	—	ND	ND	ND	ND	ND	ND	ND
Mouse hepatocytes	hep2 p10	Stably integrated	Neuroglia	Smooth muscle, cartilage	Glands, squamous epithelium	5–10%	—	ND	ND	ND	ND	ND	ND	ND
Mouse hepatocytes	hep3 p8	Stably integrated	Neuroglia	Smooth muscle	Glands, squamous epithelium	5%	—	10% EC 1+ 1% EC 2+	EC 2+/3+	30% EC 2+	NCR	NCR	Neg.	NCR
Mouse HSC	hem1 p11	Stably integrated	Neuroglia	Striated & smooth muscle, cartilage, adipose tissue	Pancreas, glands, squamous epithelium	5–10%	—	ND	ND	ND	ND	ND	ND	ND
Mouse HSC	hem2 p11	Stably integrated	Neuroglia, pigmented epithelium	Striated & smooth muscle, cartilage, adipose tissue	Pancreas, glands, squamous epithelium	5–10%	—	ND	ND	ND	ND	ND	ND	ND
Mouse HSC	hem3 p10	Stably integrated	Neuroglia, pigmented epithelium	Striated & smooth muscle, cartilage	Pancreas, liver, glands, squamous epithelium	5–10%	—	10% EC 1+	EC 3+ 5% TE 1+/2+	30% EC 2+	NCR	NCR	Striated muscle 1+	NCR

EC = embryonal carcinoma.

YST = yolk sac tumor.

ND = not done.

NCR = no expected antibody cross-reaction with murine tissues.

HSC = hematopoietic stem cells.

## References

[1] Zhang W. Y., de Almeida P. E., Wu J. C. (2008). Teratoma formation: a tool for monitoring pluripotency in stem cell research. *Current Protocols in Stem Cell Biology*.

[2] Kurman R. J., Carcangiu M. L., Herrington C. S., Young R. H. (2014). *WHO Classification of Tumours of Female Reproductive Organs*.

[3] Ben-Porath I., Thomson M. W., Carey V. J. (2008). An embryonic stem cell-like gene expression signature in poorly differentiated aggressive human tumors. *Nature Genetics*.

[4] Miura K., Okada Y., Aoi T. (2009). Variation in the safety of induced pluripotent stem cell lines. *Nature Biotechnology*.

[5] Cunningham J. J., Ulbright T. M., Pera M. F., Looijenga L. H. J. (2012). Lessons from human teratomas to guide development of safe stem cell therapies. *Nature Biotechnology*.

[6] Griscelli F., Féraud O., Oudrhiri N. (2012). Malignant germ cell-like tumors, expressing Ki-1 antigen (CD30), are revealed during in vivo differentiation of partially reprogrammed human-induced pluripotent stem cells. *The American Journal of Pathology*.

[7] Kim K., Doi A., Wen B. (2010). Epigenetic memory in induced pluripotent stem cells. *Nature*.

[8] Birraux J., Menzel O., Wildhaber B., Jond C., Nguyen T. H., Chardot C. (2009). A step toward liver gene therapy: efficient correction of the genetic defect of hepatocytes isolated from a patient with Crigler-Najjar syndrome type 1 with lentiviral vectors. *Transplantation*.

[9] Barde I., Rauwel B., Marin-Florez R. M. (2014). A KRAB/KAP1-miRNA cascade regulates erythropoiesis through stage-specific control of mitophagy. *Medecine/Sciences*.

[10] Sommer C. A., Sommer A. G., Longmire T. A. (2010). Excision of reprogramming transgenes improves the differentiation potential of iPS cells generated with a single excisable vector. *STEM CELLS*.

[11] Pasi C. E., Dereli-Öz A., Negrini S. (2011). Genomic instability in induced stem cells. *Cell Death and Differentiation*.

[12] Carey B. W., Markoulaki S., Beard C., Hanna J., Jaenisch R. (2010). Single-gene transgenic mouse strains for reprogramming adult somatic cells. *Nature Methods*.

[13] Coulouarn C., Cavard C., Rubbia-Brandt L. (2012). Combined hepatocellular-cholangiocarcinomas exhibit progenitor features and activation of Wnt and TGF*β* signaling pathways. *Carcinogenesis*.

[14] Miettinen M., Wang Z., McCue P. A. (2014). SALL4 expression in germ cell and non-germ cell tumors: a systematic immunohistochemical study of 3215 cases. *The American Journal of Surgical Pathology*.

[15] Oosterhuis J. W., Looijenga L. H. J. (2005). Testicular germ-cell tumours in a broader perspective. *Nature Reviews Cancer*.

[16] Abiko K., Mandai M., Hamanishi J. (2010). Oct4 expression in immature teratoma of the ovary: relevance to histologic grade and degree of differentiation. *The American Journal of Surgical Pathology*.

[17] Wahlström T., Henriksson M. A. (2015). Impact of MYC in regulation of tumor cell metabolism. *Biochimica et Biophysica Acta*.

[18] Ruzinova M. B., Caron T., Rodig S. J. (2010). Altered subcellular localization of c-Myc protein identifies aggressive B-cell lymphomas harboring a c-MYC translocation. *The American Journal of Surgical Pathology*.

[19] Ulbright T. M., Tickoo S. K., Berney D. M., Srigley J. R. (2014). Best practices recommendations in the application of immunohistochemistry in testicular tumors: report from the International Society of Urological Pathology consensus conference. *The American Journal of Surgical Pathology*.

[20] Zynger D. L., Dimov N. D., Luan C., Tean Teh B., Yang X. J. (2006). Glypican 3: a novel marker in testicular germ cell tumors. *The American Journal of Surgical Pathology*.

[21] van den Akker C. H. P., Schierbeek H., Rietveld T. (2008). Human fetal albumin synthesis rates during different periods of gestation. *The American Journal of Clinical Nutrition*.

[22] Horie Y., Kato M. (2000). Hepatoid variant of yolk sac tumor of the testis. *Pathology International*.

[23] Sauter G., Ebele J. N., Epstein J. I., Sesterhenn I. A. (2004). *WHO Classification of Tumours of the Urinary System and Male Genital Organs*.

[24] McIntyre A., Gilbert D., Goddard N., Looijenga L., Shipley J. (2008). Genes, chromosomes and the development of testicular germ cell tumors of adolescents and adults. *Genes Chromosomes and Cancer*.

[25] Vilette D., Emanoil-Ravier R., Tobaly J., Peries J. (1985). Studies on four cellular proto-oncogenes and their expression in PCC4 embryonal carcinoma cells: amplification of c-Ki-ras oncogene. *Biochemical and Biophysical Research Communications*.

[26] Damjanov I., Andrews P. W. (2007). The terminology of teratocarcinomas and teratomas. *Nature Biotechnology*.

[27] Kooreman N. G., Wu J. C. (2010). Tumorigenicity of pluripotent stem cells: biological insights from molecular imaging. *Journal of the Royal Society Interface*.

[28] Pierce G. B., Dixon F. J., Verney E. L. (1960). Teratocarcinogenic and tissue-forming potentials of the cell types comprising neoplastic embryoid bodies. *Laboratory Investigation*.

[29] Herszfeld D., Wolvetang E., Langton-Bunker E. (2006). CD30 is a survival factor and a biomarker for transformed human pluripotent stem cells. *Nature Biotechnology*.

[30] Yanger K., Zong Y., Maggs L. R. (2013). Robust cellular reprogramming occurs spontaneously during liver regeneration. *Genes and Development*.

[31] Almstrup K., Hoei-Hansen C. E., Wirkner U. (2004). Embryonic stem cell-like features of testicular carcinoma in situ revealed by genome-wide gene expression profiling. *Cancer Research*.

[32] Skotheim R. I., Lind G. E., Monni O. (2005). Differentiation of human embryonal carcinomas in vitro and in vivo reveals expression profiles relevant to normal development. *Cancer Research*.

[33] Rajpert-De Meyts E. (2006). Developmental model for the pathogenesis of testicular carcinoma in situ: genetic and environmental aspects. *Human Reproduction Update*.

[34] Okita K., Ichisaka T., Yamanaka S. (2007). Generation of germline-competent induced pluripotent stem cells. *Nature*.

[35] Nakagawa M., Takizawa N., Narita M., Ichisaka T., Yamanaka S. (2010). Promotion of direct reprogramming by transformation-deficient Myc. *Proceedings of the National Academy of Sciences of the United States of America*.

